# An 11-Year Retrospective Research Study of the Predictive Factors of Peri-Implantitis and Implant Failure: Analytic-Multicentric Study of 1279 Implants in Peru

**DOI:** 10.1155/2019/3527872

**Published:** 2019-06-24

**Authors:** Frank Mayta-Tovalino, Yens Mendoza-Martiarena, Percy Romero-Tapia, María Álvarez-Paucar, Luis Gálvez-Calla, Juan Calderón-Sánchez, Rodolfo Bolaños-Cardenas, Antonio Diaz-Sarabia

**Affiliations:** ^1^Faculty of Stomatology, Universidad Peruana Cayetano Heredia, Lima, Peru; ^2^Faculty of Dentistry, Universidad Nacional Mayor de San Marcos, Lima, Peru; ^3^Department of Stomatology, Centro Medico Naval–Marina de Guerra del Peru, Lima, Peru; ^4^Department of Stomatology, Instituto de Salud Oral–Fuerza Aérea del Peru, Lima, Peru

## Abstract

**Aim:**

To analyze the risk factors by logistic regression and perform the analysis of the survival rate of osseointegrated dental implants placed in public and private institutions.

**Methods:**

An analytic-multicentric study was carried out, where 1279 dental implants that were placed by specialists from January 2006 to October 2017 in public and private institutions (UPCH-SI, HCFAP, CMNAVAL, UPCH-SM, and UPSJB) were evaluated. The variables sex (X1), location (X2), hypertension (X3), antibiotic prophylaxis (X4), diabetes (X5), osteoporosis (X6), bisphosphonates (X7), history of periodontitis (X8), hypercholesterolemia (X9), bone quality (X10), bone quantity (X11), design (X12), smoker (X13), connection (X14), edentulism type (X15), staging (X16), 3D guided surgery (X17), load (X18), bone graft (X19), peri-implantitis (X20), mucositis (X21), and GBR (X22) were collected and analyzed by the Kaplan–Meier survival analysis. The logit analysis was performed among all the variables to choose the best statistical model that explains the true risk factors. The analysis was performed by multivariate logistic regression and the Kaplan–Meier test, at a level of statistical significance of *p* < 0.05.

**Results:**

It was found that the failure rate of the 1279 implants evaluated was 17.98% corresponding to only 23 implants lost as they have good longevity over time. When establishing the best multivariate logistic regression model, it was found that the variables that remained stable in relation to their statistically significant value and more stable confidence intervals were age, osteoporosis, bisphosphonates, history of periodontitis, bone quality, bone graft, connection, number of implants, GBR (guided bone regeneration), and follow-up.

**Conclusions:**

Dental implants placed by specialists in public and private institutions had a failure rate similar to that in studies previously published in other countries.

## 1. Introduction

The main challenge of oral implantology is to achieve the functionality of the implants; however, osseointegration is associated with several factors, such as the reduction of surgical trauma, the shortening of treatment time, and the improved preservation of surrounding bone and soft tissue. In cases with sufficient primary stability, the literature reports that well-planned implant placement produces high efficacy in terms of long-term success and aesthetic result [1, 2].

For this reason, the treatment with dental implants is an integral part of the patient's rehabilitation. Predictability is very important because it is a great advantage of dental implant therapy which is well documented for partially and completely edentulous patients. With the growing demand and interest on the part of the populations, it is essential to establish the optimal care and maintenance that should be given to the placed implants [3–11]. As a consequence of this situation, studies are needed to evaluate the prognosis and long-term functionality of dental implants, reporting survival rates. However, many studies describe only the survival of implants in favorable places without assuming the risk factors that could be adverse to the survival of this alternative treatment. In addition, most of the reports assessing risk factors for failure are deficient in terms of their statistical analyses since the potentials of the risk factors for failure should be determined using appropriate statistical techniques that support the statistically significant weight through the establishment of models to verify this condition [12].

In addition, survival rates do not take into account the presence of surgical, biological, and prosthetic complications; despite the remarkable survival rate of dental implants, there are an increasing number of patients with peri-implant diseases [13]. Given the possible systemic ramifications of chronic inflammation, it is essential to better understand the prevalence of peri-implant diseases and the risk factors to be able to prevent or treat this injury that affects the surrounding tissues. Currently, there is controversy regarding the therapeutic management of peri-implant diseases because they can cause various discomforts with a surgical or nonsurgical treatment which is not necessarily protocolized [14]. Therefore, these pathologies have negative impacts on systemic health or eventual loss of the implant [15]. The determination of future peri-implant diseases is necessary for clinical decision-making.

Thus, the purpose of this analytic-multicentric study was to analyze the risk factors by logistic regression and perform the analysis of the survival rate of osseointegrated dental implants placed in public and private institutions.

## 2. Materials and Methods

### 2.1. Subject Population

The study design was analytical, correlational, comparative, and retrospective. The unit of analysis comprised patients with a dental implant from the Central Hospital of the Peruvian Air Force (HCFAP), Naval Medical Center (CMNAVAL), Universidad Peruana Cayetano Heredia (UPCH) at its headquarters San Martin de Porres and San Isidro, and Universidad Privada San Juan Bautista (UPSJB). We worked with the entire population that had implants placed in public and private institutions for up to 11 years of evolution (*N* = 1279).

### 2.2. Ethical Considerations

For the execution of the study, authorization was requested to the Ethics Committee of the Universidad Peruana Cayetano Heredia approved with code SIDISI 100839, and permission was also requested to the other institutions. No risks or conflicts of interest were anticipated since the research was retrospective that used the clinical histories of the Periodontics and Implantology service of the aforementioned institutions. This research was carried out following the STROBE (Strengthening the Reporting of Observational Studies in Epidemiology) guidelines.

- Inclusion criteria:
  1. Patients of both sexes aged 18 to 80 years
  2. Patients with controlled systemic diseases
  3. Patients from hospitals of the armed forces and private universities that authorize the execution of the study
  4. Patients with clinical histories that at least have the main variables (age, sex, location of the implant, hypertension, antibiotic prophylaxis, bone quality, bone quantity, diabetes, osteoporosis, bisphosphonates, history of periodontitis, and hypercholesterolemia)
- Exclusion criteria:
  1. Patients with illegible clinical histories
  2. Patients with clinical histories that last less than 1 year or high after installation of their implants

### 2.3. Risk Factor Examinations

The risk factors associated with the failure of the implant were evaluated in the following categories: peri-implant health status (which was evaluated through the radiographic and clinical study through the probing recorded in the specialized histories of the Periodontics and Implantology service) and the variables such as sex (X1), location (X2), hypertension (X3), antibiotic prophylaxis (X4), diabetes (X5), osteoporosis (X6), bisphosphonates (X7), history of periodontitis (X8), hypercholesterolemia (X9), bone quality (X10), bone quantity (X11), design (X12), smoker (X13), connection (X14), edentulism type (X15), staging (X16), 3D guided surgery (X17), load (X18), bone graft (X19), peri-implantitis (X20), mucositis (X21), and guided bone regeneration (GBR) (X22). All these covariates were obtained from the clinical records of the institutions.

### 2.4. Statistical Analysis

To perform the descriptive statistics of the variables' risk factors, survival and failure of implants, we proceeded to obtain the univariate analysis through the measures of central tendency (mean and standard deviation) of the quantitative variables and frequencies and percentages of the qualitative variables. To determine normality, the Shapiro–Wilk test was used. Finally, the multivariate analysis was performed by logistic regression, establishing the best statistical model that explains the influence of risk factors, and then, the analysis of survival and failure of the implants was made through the Kaplan–Meier method.

## 3. Results

### 3.1. Systemic Risk Factors

When analyzing the descriptive characteristics of the systemic risk factors of the osseointegrated dental implants placed in five public and private institutions of this analytic-multicentric study, it was found that the most predominant sex was female at the UPCH-SM headquarters with 111 (50.9%), while the location was primarily presented in the mandible at the CMNAVAL headquarters with 77 (37.3%); however, in all institutions, an antibiotic prophylaxis protocol (413 (100%)) was applied. In relation to diabetes and osteoporosis, it was presented mostly at the UPCH-SM headquarters with 3 (20%) and 1 (1.8%), respectively. On the contrary, the presence of bisphosphonate consumption was only found in 5 (100%) of patients at the UPCH-SI headquarters. Finally, the history of periodontitis and hypercholesterolemia was increased at CMNAVAL with 49 (43.3%) and 21 (50%), respectively (Table 1).

### 3.2. Surgical Risk Factors

It was found that the predominant bone quality was type II (132 (38.2%)), the bone quantity was type B (82 (33.7%)), the hybrid design was the most prevalent (123 (43.6%)), and the Morse connection was the most used (116 (55.5%)). However, the variables smoking habit, 3D guided surgery, type of edentulism, and type of prosthetic load had low prevalences in most institutions. Otherwise, bone grafts and GBR were more prevalent at UPCH-SI (86 (6.4%)) and CMNAVAL (46 (36.5%)), respectively (Table 2).

### 3.3. Multivariate Logistic Regression Model

When establishing the best multivariate logistic regression model to analyze the influence of each risk factor on the success and survival of osseointegrated implants, it was found that the variables that remained stable in relation to their statistically significant value and more stable confidence intervals were age, osteoporosis, bisphosphonates, history of periodontitis, bone quality, bone graft, connection, number of implants, GBR, and follow-up. However, only the variables age, osteoporosis, history of periodontitis, bone quality, number of implants, GBR, and follow-up had ORs really considered as risk factors (Table 3).

### 3.4. Implant Failure Rate and Implant Survival Rate

It was found that the failure rate of the 1279 implants evaluated was 17.98% corresponding to only 23 implants lost, so it can be inferred that the implants are a good treatment alternative given that they have good longevity over time (Table 4). The evaluation of the 11-year cumulative survival of the osseointegrated implants showed that the survival rate was inversely proportional to time in years. During the first and the second year, a rate of 99.4% was found, while at eleven to twelve years, it was reduced to 37.8% (Table 5).

### 3.5. Distribution of Implant Survival according to Headquarters

**Figure 1:**
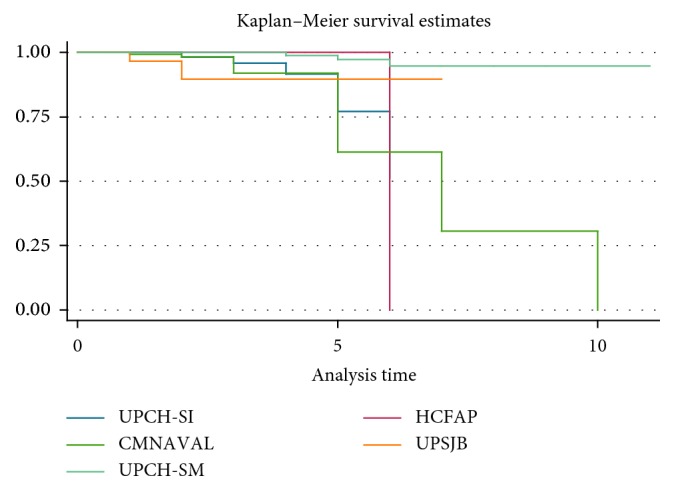
Survival rate according to the time for the five institutions.

Finally, quantifying the survival rate of the osseointegrated implants for the five institutions evaluated in this multicentric study showed that, in all the institutions (UPCH-SI, HCFAP, CMNAVAL, UPSJB, and UPCH-SM), the rate was above 96.5%; however, the survival rate of dental implants continues to decrease progressively as the years of functionality in the oral cavity increase (Table 6) (Figure 1).

## 4. Discussion

In recent years, there is a great demand from patients who are treated by dental implants to rehabilitate the edentulous areas. Therefore, the effect of certain factors on the loss of implants, which generate a greater risk of predisposing to marginal peri-implant bone loss, should be considered. To evaluate the survival of the implants, it is necessary to identify the presence of complications. If reported, the time of implant loss is described as early (before prosthetic loading) or late (after prosthetic loading). It is agreed that survival is expressed in cumulative survival rates, where a successful implant refers to the presence of an implant in the absence of both biological and prosthetic complications [15].

The currently accepted dental implant planning protocol includes clinical examination, diagnostic tests, and clinical history, to establish treatment options, and an appropriate maintenance phase. The placement of the implants is conditioned to the usual restrictions for minor surgery that are established by the systemic conditions of the patient. It should be noted that these care protocols vary according to the work philosophy that is handled in each institution; therefore, the level of evidence should help identify possible risk factors that act as contraindications for implant therapy [16]. There is little literature that has analyzed the effect of multiple risk factors on implant survival, especially in elderly patients who have some chronic systemic disease, have a history of smoking, and consume a drug that could compromise osseointegration.

Consequently, with the results of this investigation, it was found that the long-term survival rate of the implants is a tool for objective measurement of the stability of the implants which is 99.4%, which was similar to that described by Cosyn et al. [17] (96.5%), Balshi et al. [18] (99.8%), and Moreira Melo et al. [19] (92.65%). The overall success rate was above 95%, which is in agreement with other studies that reported values ranging from 81% to 93% [20–22]. A clear example of an investigation that confirms through a multivariate model similar results is that described by Borba et al. [23] who found that the survival rate was 91.8%. The multivariate GEE analysis revealed that a significant risk factor for the implant failure was the implant in the maxillary area (*p* = 0.014), and the bone graft seemed to be a risk factor for implant failure (*p* = 0.054). GEE analyses showed that maxillary implants had significantly worse results in this population and were considered a risk factor for implant failure. Therefore, our results suggested that implants placed in an area of bone augmentation, age, bone quality, and GBR had a tendency to fail, as demonstrated by a logit model.

Otherwise, in relation to the failure of the implants, according to the results described by Mohajerani et al. [24], of the 1093 implants evaluated, only 73 cases (6.68%) failed during the early stages of osseointegration. The groups were significantly different in terms of implant surface, placement in a new alveolus, use of an antibiotic prophylactic, and bone density (*p* < 0.05). However, age, sex, height of the implant, type of the implant (cylindrical or conical), and placement in one or two stages were not significantly different between the two groups (*p* < 0.05). These results differ from the data obtained in the present study given that if they were found in relation to their statistically significant value and more stable confidence intervals in the factors age, osteoporosis, bisphosphonates, history of periodontitis, bone quality, bone graft, connection, number of implants, GBR, and follow-up (*p* < 0.05). Probably, the discrepancies can be attributed to certain variables that are difficult to control such as diet, race, and hygiene habits that may be influencing the survival of dental implants.

On the contrary, when looking for scientific evidence, no literature was found that mathematically established a regression model on the risk factors of dental implants in Peru. Therefore, the present investigation opens a large line of research because by statistically determining these factors, the prevalence of biological complications of osseointegrated implants can be reduced. Public and private institutions would probably have a great acceptance in rethinking their surgical and prosthetic protocols when planning the placement of dental implants and thus be able to provide a therapy that has high predictability and thus guarantee a success rate of dental implants in Peru.

The biggest limitation of this research was that the current literature has been evaluated on the risk factors for implants without necessarily establishing a regression model that statistically endorses the risk factors that actually significantly influence the failure of the implants. In addition, most of the research is in association with many confounding factors; numbers of subcategories can often also vary a statistically significant comparison, and the follow-up of the implants in the times varies and is often short-term. There are many risk factors that are potential and require that the clinician have a wide knowledge and understanding of these factors to discuss them with each patient and consider them in the planning and treatment of dental implants.

Another limitation of this study was that it only retrospectively evaluated some predictors such as age, sex, type of the implant, surface, length, diameter, location, bone quality, and bone quantity that could influence the survival rate among various factors that are related, in comparison with other investigations, with the survival of the implant. In addition, there were several factors related to systemic diseases that could influence the survival rate. Finally, several factors combined could determine the survival rate, but they were not analyzed in this study. It is recommended to carry out well-controlled long-term prospective studies, which confirm the relationship between the factors that influence the survival rate in order to be analyzed consecutively. When many investigations draw a common conclusion about the reason for implant failure, common factors related to the survival rate will have a greater influence on failure; therefore, more studies are needed on them. For this reason, it is suggested to perform long-term longitudinal studies that complement the evidence on which are the true risk factors that continue to influence the failure of dental implants in a Peruvian population.

## 5. Conclusions

In conclusion, it was found that, in the 1279 osseointegrated implants of this multicentric study, when establishing the best multivariate logistic regression model, the variables that remained stable in relation to their statistically significant value and confidence intervals were age, osteoporosis, bisphosphonates, history of periodontitis, bone quality, bone graft, connection, number of implants, GBR, and follow-up. In addition, it was shown that the failure rate was only 17.98% corresponding to only 23 implants lost, observing that the survival rate is inversely proportional to time in years and reporting that, during the first and the second year, a rate of 99.4% was found, while at eleven to twelve years, it was reduced to 37.8%. Finally, it was shown that, in all the institutions (UPCH-SI, HCFAP, CMNAVAL, UPSJB, and UPCH-SM), the success rate was above 96.5%.

## Figures and Tables

**Table 1:** General risk factors of osseointegrated dental implants.

		Public and private institutions					
Factors	Category	UPCH-SI, *n* (%)	HCFAP, *n* (%)	CMNAVAL, *n* (%)	UPSJB, *n* (%)	UPCH-SM, *n* (%)	Total, *n* (%)
Sex (X1)	Female	50 (22.9)	15 (6.8)	29 (13.3)	13 (5.9)	111 (50.9)	218 (100)
	Male	17 (7.9)	26 (12.2)	114 (53.5)	16 (7.5)	40 (18.7)	213 (100)

Location (X2)	Jaw	27 (13.1)	21 (10.19)	77 (37.3)	15 (7.2)	66 (32.0)	216 (100)
	Maxilla	40 (17.7)	20 (8.8)	66 (29.3)	14 (6.2)	85 (37.7)	225 (100)

Hypertension (X3)	Not present	53 (14.6)	28 (7.7)	124 (34.1)	23 (6.3)	135 (37.1)	363 (100)
	Present	14 (20.5)	13 (19.1)	19 (27.9)	6 (8.8)	16 (23.5)	68 (100)

Antibiotic therapy (X4)	Not present	0	0	0	0	0	0
	Present	67 (15.5)	41 (9.5)	143 (33.1)	29 (6.7)	151 (35.0)	431 (100)

Diabetes (X5)	Not present	67 (15.9)	41 (9.7)	138 (32.7)	27 (6.4)	148 (35.1)	421 (100)
	Present	0	0	5 (50)	2 (20)	3 (20)	10 (100)

Osteoporosis (X6)	Not present	57 (13.6)	40 (9.5)	143 (34.1)	29 (6.9)	150 (35.8)	419 (100)
	Present	10 (83.3)	1 (8.3)	0	0	1 (8.3)	12 (100)

Bisphosphonates (X7)	Not present	62 (14.5)	41 (9.6)	143 (33.5)	29 (6.8)	151 (35.4)	426 (100)
	Present	5 (100)	0	0	0	0	5 (100)

History of periodontitis (X8)	Not present	63 (19.8)	21 (6.6)	94 (29.5)	17 (5.2)	123 (38.6)	318 (100)
	Present	4 (3.5)	20 (17.7)	49 (43.3)	12 (10.6)	28 (24.7)	113 (100)

Hypercholesterolemia (X9)	Not present	67 (17.2)	40 (10.2)	122 (31.3)	23 (5.9)	137 (35.2)	369 (100)
	Present	0	1 (2.3)	21 (50)	6 (14.2)	14 (33.3)	42 (100)

UPCH-SI: Universidad Peruana Cayetano Heredia, San Isidro; HCFAP: Hospital Central de la Fuerza Aérea del Perú; CMNAVAL: Centro Medico Naval; UPSJB: Universidad Privada San Juan Bautista; UPCH-SM: Universidad Peruana Cayetano Heredia, San Martin de Porres.

**Table 2:** Surgical risk factors of osseointegrated dental implants.

Factors	Category	UPCH-SI, *n* (%)	HCFAP, *n* (%)	CMNAVAL, *n* (%)	UPSJB, *n* (%)	UPCH-SM, *n* (%)	Total, *n* (%)
Bone quality (X10)	Type I	2 (7.4)	0	7 (25.9)	1 (3.7)	17 (62.9)	27 (100)
	Type II	36 (10.4)	32 (9.8)	119 (34.4)	26 (7.5)	132 (38.2)	345 (100)
	Type III	28 (49.1)	9 (15.7)	17 (29.8)	2 (3.5)	1 (1.7)	57 (100)
	Type IV	1 (50)	0	0	0	1 (50)	2 (100)

Bone quantity (X11)	A	0	1 (4.5)	6 (27.2)	1 (4.5)	14 (63.6)	22 (100)
	B	47 (19.3)	33 (13.5)	68 (27.9)	13 (5.3)	82 (33.7)	243 (100)
	C	18 (12.1)	7 (4.7)	69 (46.6)	14 (9.4)	40 (27.0)	148 (100)

Design (X12)	Conical	6 (7.32)	0	38 (46.3)	27 (32.9)	11 (13.4)	82 (100)
	Cylindrical	20 (29.8)	14 (20.9)	16 (23.8)	0	17 (25.3)	67 (100)
	Hybrid	41 (14.5)	27 (9.5)	89 (31.5)	2 (0.7)	123 (43.6)	282 (100)

Smoker (X13)	Not present	60 (14.1)	41 (9.6)	143 (33.7)	29 (6.8)	151 (35.6)	424 (100)
	Present	7 (100)	0	0	0	0	7 (100)

Connection (X14)	Internal	7 (3.2)	40 (18.6)	130 (60.4)	3 (1.4)	35 (16.2)	215 (100)
	External	6 (85.7)	0	18 (14.2)	0	0	7 (100)
	Morse cone	54 (25.8)	1 (0.4)	12 (5.7)	26 (12.4)	116 (55.5)	209 (100)

Type of edentulism (X15)	Total	3 (21.4)	0	0	0	11 (78.5)	14 (100)
	Partial	64 (15.3)	41 (9.8)	143 (34.2)	29 (6.9)	140 (33.5)	417 (100)

Staging (X16)	First stage	1 (9.0)	0	3 (27.2)	6 (54.5)	1 (9.0)	11 (100)
	Second stage	66 (15.7)	41 (9.7)	140 (33.3)	23 (5.4)	150 (35.7)	420 (100)

3D surgery (X17)	Not present	67 (15.5)	41 (9.5)	143 (33.1)	29 (6.7)	151 (35.0)	431 (100)
	Present	0	0	0	0	0	0

Load (X18)	Early	0	0	7 (41.1)	6 (35.2)	4 (23.5)	17 (100)
	Conventional	67 (16.1)	41 (9.9)	136 (32.8)	23 (5.5)	147 (35.5)	414 (100)

Bone graft (X19)	Not present	59 (19.2)	35 (11.4)	95 (31.0)	14 (4.5)	103 (33.6)	306 (100)
	Present	86 (6.4)	6 (4.8)	48 (38.4)	15 (12.0)	48 (38.4)	125 (100)

Peri-implantitis (X20)	Not present	59 (14.4)	40 (9.8)	134 (32.8)	27 (6.6)	148 (36.2)	408 (100)
	Present	8 (34.7)	1 (4.3)	9 (39.1)	2 (8.7)	3 (13.0)	23 (100)

Mucositis (X21)	Not present	58 (14.2)	40 (9.8)	135 (33.0)	27 (6.6)	146 (36.2)	408 (100)
	Present	9 (39.1)	1 (4.3)	8 (34.7)	2 (8.7)	3 (13.0)	23 (100)

GBR (X22)	Not present	51 (16.7)	34 (11.1)	97 (31.6)	16 (5.2)	107 (35.0)	305 (100)
	Present	16 (12.7)	7 (5.5)	46 (36.5)	13 (10.3)	44 (34.9)	126 (100)

**Table 3:** Multivariate logistic regression model of each risk factor on the success and survival of osseointegrated implants.

Independent variables	OR	*p*	95% CI
Age (X0)	1.0	0.122	0.98–1.10
Sex (X1)	0.9	0.879	0.23–3.48
Location (X2)	0.5	0.312	0.13–1.88
Hypertension (X3)	0.5	0.463	0.11–2.72
Antibiotic therapy (X4)	—	—	—
Diabetes (X5)	5.6	0.167	0.48–65.9
Osteoporosis (X6)	44.8	0.011	2.40–834.8
Bisphosphonates (X7)	0.09	0.205	0.00–3.65
History of periodontitis (X8)	3.1	0.082	0.86–11.31
Hypercholesterolemia (X9)	5.1	0.046	1.02–25.44
Bone quality (X10)	5.8	0.021	1.30–26.6
Bone quantity (X11)	0.5	0.268	0.15–1.69
Design (X12)	1.5	0.388	0.57–4.16
Smoker (X13)	1.6	0.798	0.04–60.4
Connection (X14)	0.4	0.038	0.22–0.95
Type of edentulism (X15)	0.4	0.527	0.03–5.26
Staging (X16)	—	—	—
3D surgery (X17)	—	—	—
Load (X18)	—	—	—
Bone graft (X19)	0.1	0.183	0.01–2.16
GBR (X22)	24.1	0.007	2.34–249.6
Follow-up (X23)	1.7	0.000	1.35–2.31
Number of implants (X24)	1.1	0.290	0.90–1.39

OR: odds ratio; CI: confidence interval; GBR: guided bone regeneration. Logit model: all the variables were entered in the statistical analysis of the multivariate model. The logit model showed that age, sex, location of the implant, antibiotic therapy, diabetes, bisphosphonates, history of periodontitis, bone quantity, implant design, smoking habit, type of edentulism, bone graft, and number of implants placed were not factors of statistically significant risk in the general logistic model for the failure of the implants (*p* < 0.05).

**Table 4:** Rate of failure of osseointegrated implants in eleven years.

	Implants	Lost	Failure rate (%)	95% CI
Failure	1279	23	17.98	11.95–27.06

**Table 5:** Evaluation of the cumulative survival in 11 years of osseointegrated implants.

Time (years)	Implants	Failure	Survival rate (%)	95% CI
1-2	431	2	99.4	97.8–99.8
2-3	320	3	98.3	96.0–99.3
3-4	217	5	95.4	91.5–97.6
4-5	117	2	93.6	88.5–96.4
5-6	88	5	87.0	78.6–92.2
6-7	49	4	78.9	67.2–86.8
7-8	33	1	75.7	62.5–84.8
8-9	16	0	75.7	62.5–84.8
9-10	5	0	75.7	62.5–84.8
10-11	2	1	37.8	1.6–79.3
11-12	1	0	37.8	1.6–79.3

The Kaplan–Meier method is used to determine the survival rate of the implant. The analysis was performed on 1279 osseointegrated implants during 11 years of functionality.

**Table 6:** Distribution of implant survival by headquarters.

Institution	Time (years)	Implants	Failure	Survival rate (%)	95% CI
UPCH-SI	1	67	0	100	—
	2	56	1	98.2	87.9–99.7
	3	40	1	95.7	83.8–98.9
	4	23	1	91.6	74.4–97.4
	5	19	3	77.1	54.3–89.5
	6	6	2	51.4	19.0–76.5

HCFAP	1	41	0	100	—
	2	14	0	100	—
	3	7	0	100	—
	4	4	0	100	—
	6	1	1	0.00	—

CMNAVAL	1	143	1	99.3	95.1–99.9
	2	93	1	98.2	92.9–99.57
	3	62	4	91.9	82.3–96.3
	4	4	0	91.9	82.3–96.3
	5	3	1	61.2	82.3–96.3
	7	2	1	30.6	80.0–90.9
	10	1	1	0.00	1.0–73.6

UPSJB	1	29	1	96.5	77.9–99.5
	2	14	1	89.6	62.1–97.5
	3	4	0	89.6	62.1–97.5
	7	1	0	89.6	62.1–97.5

UPCH-SM	1	151	0	100	—
	2	143	0	100	—
	3	104	0	100	—
	4	85	1	98.8	91.9–99.8
	5	64	1	97.2	89.0–99.3
	6	39	1	94.7	83.8–98.3
	7	30	0	94.7	83.8–98.3
	8	15	0	94.7	83.8–98.3
	9	4	0	94.7	83.8–98.3
	11	1	0	94.7	83.8–98.3

## Data Availability

The data used to support the findings of this study are available from the corresponding author upon request.
